# Comparison of the right–left ventricular stroke volume difference evaluated by echocardiography in patients with chronic heart failure complicated with cardiogenic pulmonary edema and pneumonia

**DOI:** 10.3389/fcvm.2025.1693941

**Published:** 2025-12-05

**Authors:** Yaru Yan, Haotian Zhao, Jiapeng He, Li Li, Heling Zhao, Yuquan Ye

**Affiliations:** 1 Department of Ultrasound Medicine, Hebei Medical University, Shijiazhuang, Hebei, China; 2 Department of Ultrasound, Shijiazhuang People’s Hospital, Shijiazhuang, Hebei, China; 3 Department of Ultrasound, Hebei General Hospital, Shijiazhuang, Hebei, China; 4 Department of 4th Cardiovascular, Hebei General Hospital, Shijiazhuang, Hebei, China; 5 Department of Critical Care Medicine, Hebei General Hospital, Shijiazhuang, Hebei, China

**Keywords:** cardiogenic pulmonary edema, chronic heart failure, stroke volume, lung ultrasound, echocardiography

## Abstract

**Background:**

Although B-lines in lung ultrasound may result from diverse etiologies, the presence of left ventricular enlargement and reduced left ventricular ejection fraction (LVEF) generally supports their origin in cardiogenic pulmonary edema (CPE). However, these sonographic findings can also occur in patients with chronic heart failure (CHF) concurrent pneumonia, potentially leading to inappropriate clinical decisions of fluid removal. This study aimed to investigate the diagnostic value of echocardiography-derived right ventricular stroke volume (RVSV) and left ventricular stroke volume (LVSV) difference (ΔSV) in differentiating CPE from pneumonia among CHF patients with acute dyspnea.

**Methods:**

This retrospective observational study enrolled CHF patients presenting with acute dyspnea, subsequently classified as either CPE or pneumonia based on comprehensive diagnostic evaluation. The diagnosis was established by attending physicians through integrated assessment of pulmonary imaging, laboratory biomarkers, and clinical examination findings. Additionally, 30 asymptomatic CHF patients without dyspnea were included as controls. Standard echocardiographic measurements included RVSV, LVSV, tricuspid annular plane systolic excursion (TAPSE) and mitral annular plane systolic excursion (MAPSE), and the ΔSV (difference between RVSV and LVSV) and the ratio of TAPSE to MAPSE (TAPSE/MAPSE) were calculated, respectively. The average value of the ratio between the early diastolic peak velocity of the mitral valve and the diastolic peak velocity of the septal/lateral (E/e′) was considered as the left ventricular filling pressure.

**Results:**

Among 133 CHF patients with acute dyspnea in the study, 58 had CPE. Between CHF-CPE group and CHF-pneumonia group, the ROC analysis showed that the area under the curve (AUC) of ΔSV was 0.772 (sensitivity 67.24%, specificity 78.67%), and of TAPSE/MAPSE was 0.724 (sensitivity 53.45%, specificity 82.67%). Between CHF-CPE group and CHF-conrtol group, the AUC of ΔSV was 0.830 (sensitivity 87.93%, specificity 76.67%), and of TAPSE/MAPSE was 0.656 (sensitivity 53.45%, specificity 80.00%). Multivariate logistic regression analysis showed that ΔSV was an independent influencing factor, whether between the CHF-CPE group and the CHF-pneumonia group (odds ratio = 1.076, 95% CI: 1.019–1.137), or between the CHF-CPE group and the CHF-conrtol group (odds ratio = 1.066, 95% CI: 1.007–1.129).

**Conclusions:**

In CHF patients with acute dyspnea, the difference between RVSV and LVSV measured by echocardiography is helpful in distinguishing CPE from pneumonia. Nevertheless, further investigation with a larger cohort is necessary to confirm our conclusion.

## 1 Introduction

Heart failure is a clinical syndrome characterized by structural (e.g., cardiac enlargement, myocardial hypertrophy) and/or functional (impaired systolic or diastolic function) abnormalities of the heart. These changes result from primary cardiac causes or various secondary factors that lead to hemodynamic overload and myocardial decompensation. In the United States, approximately 6.2 million adults are affected by heart failure (1). Chronic heart failure (CHF) refers to a persistent state of cardiac dysfunction where compromised heart function cannot adequately support routine daily activities. Patients may experience stable symptoms, gradual deterioration, or acute decompensation (2). While CHF patients maintain sufficient organ perfusion and oxygenation at rest, their cardiac function is invariably impaired to varying degrees. To compensate for reduced cardiac output, CHF typically induces left ventricular (LV) remodeling and dilatation—processes that help restore cardiac output to near-normal levels (3). Acute dyspnea secondary to CHF constitutes a primary reason for hospitalization. The most critical precipitating factors include worsening heart failure symptoms and the development of cardiogenic pulmonary edema (CPE). CPE occurs when LV dysfunction creates a mismatch in fluid dynamics, leading to pulmonary circulatory congestion and subsequent fluid extravasation into pulmonary interstitium and alveoli.

Impaired LV function is widely recognized as the primary contributor to CPE development. Notably, LV diastolic dysfunction appears to play a more significant role than systolic dysfunction, as evidenced by studies on mechanical ventilation weaning-induced pulmonary edema (4, 5). However, beyond LV involvement, right ventricular (RV) function also critically influences acute pulmonary edema pathogenesis. Although elevated LV filling pressure and left atrial pressure directly drive pulmonary edema formation, these pressures are fundamentally sustained by RV-generated hemodynamic forces (3). A key mechanism underlying pulmonary fluid accumulation is the mismatch between RV stroke volumes (RVSV) and LV stroke volumes (LVSV) (6), where excessive RV output relative to LV capacity leads to pulmonary circulatory congestion. Despite this understanding, few studies have quantitatively assessed whether RV-LV volumetric mismatch correlates with pulmonary edema occurrence in CHF patients. Further research is needed to clarify this relationship and its clinical implications.

Pneumonia represents another critical etiology of acute respiratory failure in patients with CHF. Among CHF cases requiring emergency care due to cardiac decompensation, respiratory infections (including pneumonia) account for 35.4%, whereas pulmonary edema alone contributes to 11.3% (7). Lung ultrasound (LUS) serves as a highly sensitive tool for detecting pulmonary edema in CHF patients, as even minimal extravascular lung water generates observable B-lines. However, B-lines are not pathognomonic for pulmonary edema—they can also manifest in pneumonia, particularly interstitial pneumonitis. In CHF patients with concurrent pneumonia, echocardiography and LUS findings may be misleading. The presence of impaired LV function and typical heart failure signs could lead clinicians to erroneously attribute B-lines solely to pulmonary edema, overlooking the coexisting pulmonary infection. Misinterpreting B-lines as purely CPE may prompt overzealous diuresis, potentially resulting in excessive volume depletion and subsequent hypovolemic shock.

Bedside echocardiography serves as a readily available, non-invasive, and convenient method for hemodynamic monitoring. Echocardiographically measured LVSV and derived cardiac output demonstrate good correlation with pulse indicator continuous cardiac output (PiCCO) measurements (8), making this technique particularly valuable for rapid assessment of stroke volume and cardiac output in emergency settings. We hypothesize that CPE results from a mismatch between RVSV and LVSV, whereas CHF patients with pneumonia (without pulmonary edema) maintain balanced ventricular outputs. To test this hypothesis, we employed echocardiography to measure RVSV and LVSV, calculating their differential value (ΔSV) to investigate potential disparities between CHF patients with CPE and those with pneumonia.

## 2 Materials and methods

### 2.1 Study population

This retrospective observational study included patients with CHF and acute dyspnea admitted to a tertiary hospital from March 1, 2023 to May 30, 2024 as the observation subjects. The study was performed according to the principles of the Declaration of Helsinki. This study was was approved by the Ethics Committee of our hospital (No. 2024–167) and registered at the China Clinical Trial Registration Centre (No. ChiCTR2400085662). All point-of-care ultrasound examinations were performed as part of routine clinical practice upon clinical physician request, with prior informed consent obtained from patients or their legal guardians.

**Figure 1:**
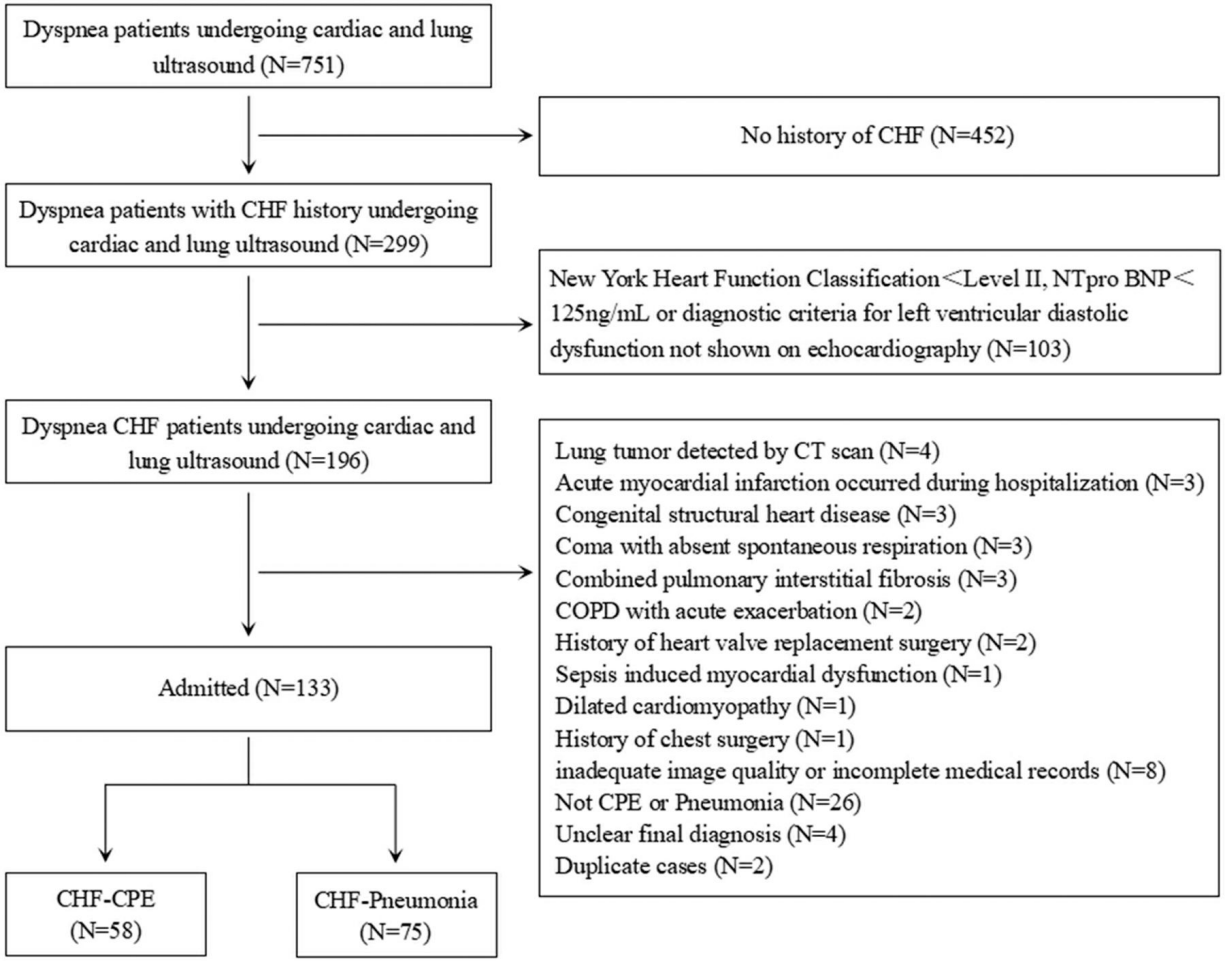
Flowchart of the study. CHF: chronic heart failure; COPD: chronic obstructive pulmonary disease; CPE: cardiogenic pulmonary edema.

Our emergency and critical care ultrasound team, composed of ultrasound physicians, is responsible for performing bedside ultrasonography for acute cardiopulmonary complications and respiratory distress across all clinical departments. From the cohort of patients who underwent cardiopulmonary ultrasound for acute dyspnea, we selected those meeting established CHF diagnostic criteria for further analysis (Figure 1).

The inclusion criteria were as follows: (1) confirmed CHF diagnosis with a documented history ≥3 months; (2) New York Heart Association (NYHA) functional class ≥II, with admission laboratory findings of either brain natriuretic peptide (BNP) ≥35 ng/L or N-terminal pro-B-type natriuretic peptide (NT-proBNP) ≥125 ng/L, along with echocardiographic evidence of LV diastolic dysfunction ≥grade I; (3) acute dyspnea meeting at least one of the following: subjective dyspnea or orthopnea, respiratory rate ≥20 breaths/min; oxygen saturation ≤93%, oxygenation index ≤300 mmHg; (4) pulmonary infiltrative shadows on chest CT or chest x-ray, or moist crackles on lung auscultation.

Exclusion criteria: (1) age <18 years; (2) structural heart disease (e.g., congenital defects, vascular anomalies, dilated/hypertrophic cardiomyopathy, or left ventricular noncompaction); (3) acute coronary syndrome (including myocardial infarction); (4) persistent atrial fibrillation or paroxysmal atrial fibrillation attack period; (5) moderate-to-large pericardial effusion; (6) chronic interstitial fibrosis, pneumothorax, subcutaneous emphysema, lung tumors, or prior thoracic/mediastinal surgery; (7) unclear etiology of dyspnea after clinical evaluation; (8) inability to undergo cardiopulmonary ultrasound or poor image quality.

The etiological diagnosis of dyspnea was jointly interpreted by one cardiovascular physician and one critical care physician based on clinical manifestations, electrocardiogram, cardiopulmonary imaging, laboratory tests (including NT-proBNP, complete blood count, myocardial enzyme markers, C-reactive protein, procalcitonin, bacterial culture, etc.), medical record review, and treatment response. The diagnosis of CPE was established based on a combination of the following: (1) typical clinical manifestations, such as orthopnea and bilateral pulmonary rales on auscultation; (2) supportive findings from laboratory tests, and imaging studies demonstrating pulmonary infiltrates or cardiomegaly on chest x-ray or CT and reduced ejection fraction on echocardiography; and (3) a positive response to diuretic therapy, evidenced by the alleviation of symptoms following administration. The diagnosis of pneumonia was established based on clinical symptoms (e.g., fever, cough, purulent sputum), radiographic evidence of new pulmonary infiltrates on chest imaging. The diagnoses of CPE and pneumonia were rigorously established in accordance with prevailing international guidelines (9, 10), supplemented by a consensus from in-hospital senior specialists to ensure diagnostic accuracy in complex cases. Patients who met the criteria for CHF with CPE were enrolled in the CHF combined with CPE (CHF-CPE) group, those with significant acute pulmonary edema in both lungs accompanied by minor localized pulmonary inflammation could also be included in the CHF-CPE group after evaluation. CHF patients without pulmonary edema but presenting with dyspnea due to pneumonia, and in whom CPE episodes were ruled out, were assigned to the CHF combined with pneumonia (CHF-pneumonia) group. Cases concurrently exhibiting both conditions with indistinguishable disease severity were excluded.

Additionally, 30 patients with a documented history of CHF for more than 3 months who were in a non-heart failure exacerbation period (during routine physical examination or outpatient follow-up) were selected. Inclusion criteria: (1) NYHA functional class ≥II without apparent heart failure symptoms at the time of visit; (2) stable respiratory and circulatory function with no manifestations of dyspnea; (3) normal ranges of inflammatory markers and myocardial enzyme indicators in laboratory tests; (4) LUS showing no significant pulmonary exudative manifestations or only 1–2 B-lines (considered due to gravitational factors) at one or both lung bases. Exclusion criteria were the same as those for the observation group mentioned above.

### 2.2 Echocardiography

The ultrasound examination was performed using Philips EPIQ7 or Philips CX50 (Philips ultrasound diagnostic system, Netherlands), with a phased-array S5-1 probe (1–5 MHz) for cardiac ultrasound. The sonographers and image analysts were blinded to the clinical diagnoses. According to the guidelines Echocardiography (11), the following cardiac ultrasound parameters were measured: LV ejection fraction (LVEF), LV end-diastolic volume (LVEDV), RV diameter (RVD), right atrial diameter (RAD), tricuspid annular plane systolic excursion (TAPSE), mitral annular plane systolic excursion (MAPSE), tricuspid regurgitation velocity (TRV), pulmonary artery systolic pressure (PASP), peak systolic velocity of RV free wall tissue (RV-s'), early and late diastolic peak flow velocities of the mitral valve (E and A), and peak diastolic velocities of the mitral annulus at the septal and lateral walls (septal e′ and lateral e′). The E/A and E/e′ was also calculated.

In the apical four-chamber view, the left atrial area (LAA) was traced at end-diastole and end-systole, including the maximum LAA (LAAmax) and minimum LAA (LAAmin), to calculate the variation of LAA (LAA-V) using the formula: LAA-V = (LAAmax—LAAmin)/LAAmax. Due to the need for urgent treatment in patients with acute dyspnea, bedside ultrasound examination time was limited, making it impractical to measure LA volume. Based on our experience, using single-plane fractional area change may be meaningful.

The long-axis view of the inferior vena cava (IVC) was obtained in the subxiphoid sagittal plane. A segment of the IVC with significant respiratory variation, located 1–3 cm from the right atrial junction, was selected as the measurement site. The end-expiratory diameter of the IVC (IVCD) was measured, and the degree of IVC collapse during inspiration was observed to calculate the variation rate of the inferior vena cava (IVC-V) using the formula: IVC-V = (IVCD—IVCD at end-inspiration)/IVCD. For patients receiving non-invasive mechanical ventilation, measurements were taken after briefly discontinuing ventilatory support or reducing ventilator settings. The original ventilator parameters were immediately restored after obtaining stable ultrasound images to minimize the impact of mechanical ventilation on IVC measurements.

**Figure 2:**
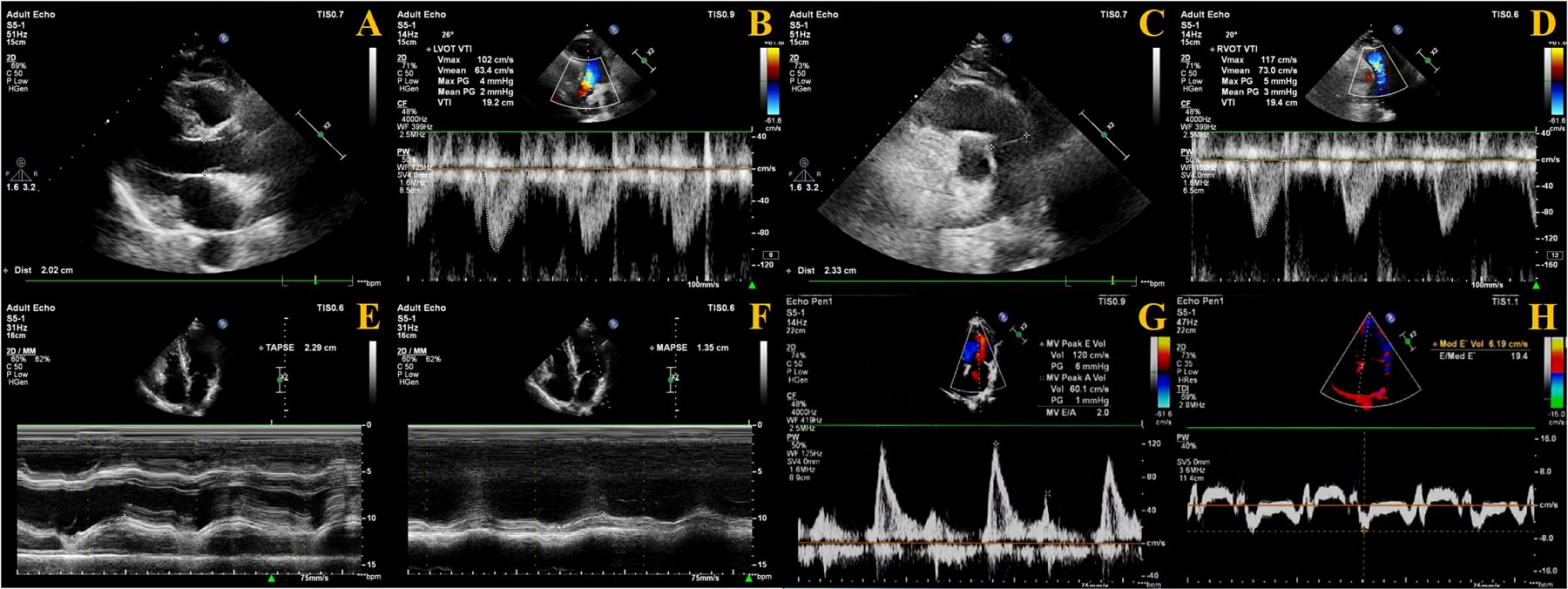
Measurement methods of echocardiography. **(A)** LVOT diameter measurement; **(B)** LVOT-VTI measurement; **(C)** ROVT diameter measurement; **(D)** RVOT-VTI measurement; **(E)** TAPSE measurement; **(F)** MAPSE measurement; **(G)** E velocity measurement; **(H)** Septal e′ measurement.

The velocity time integral (VTI) of both the LV outflow tract (LVOT) and RV outflow tract (RVOT) must be measured. Using color Doppler mode, blood flow from the LVOT toward the aorta and from the RVOT toward the pulmonary artery was visualized. The sample line was aligned with the direction of blood flow, and pulsed-wave Doppler mode was used to acquire images for measuring LVOT-VTI and RVOT-VTI, respectively. The diameter of the LVOT and RVOT was measured at the aortic valve annulus and pulmonary valve annulus, respectively. The fomula as follows (Figure 2):

1. Cross-sectional area (CSA): CSA = π × (diameter/2)^2^.
2. Left ventricular stroke volume (LVSV): LVSV = (VTI × CSA) of LVOT.
3. Right ventricular stroke volume (RVSV): RVSV = (VTI × CSA) of RVOT.
4. The difference between RVSV and LVSV is expressed as Delta SV (ΔSV): ΔSV = RVSV—LVSV.

Regarding cardiac coupling ultrasound parameters, ΔSV represents the difference in stroke volume between the RV and LV per cardiac cycle. Based on the study by Zhang et al. (12), we selected TAPSE and MAPSE—parameters reflecting the longitudinal systolic function of the RV and LV, respectively—and calculated their ratio (TAPSE/MAPSE) (Figure 2).

### 2.3 LUS examination

In this study, LUS was performed using a convex C5-1 probe (1–5 MHz) for transthoracic pulmonary scanning. A 12-area scanning protocol was adopted: the anterior and lateral chest walls were examined in supine or sitting position, while the posterior chest was examined in sitting or lateral decubitus position. Using the parasternal line, anterior axillary line, posterior axillary line, and paravertebral line as vertical boundaries, and the nipple level as the horizontal boundary, the lung ultrasound zones were divided into upper and lower regions of the anterior chest, lateral chest, and posterior chest, totaling 12 areas.

Each lung area was scored based on sonographic patterns: (1) 0-point: A-lines or ≤2 B-lines, indicating normal aeration; (2) 1-point: ≥3 discrete, well-defined B-lines, suggesting interstitial edema at the interlobular septal level; (3) 2-point: confluent B-lines with blurred margins, indicating alveolar-interstitial edema; (4) 3-point: lung consolidation with air bronchograms or shred sign, with or without pleural effusion, suggesting extensive alveolar collapse and loss of ventilation. The LUS score was calculated by summing the scores from all 12 areas.

### 2.4 Other parameters evaluated

We collected demographic and clinical data for each patient, including age, gender, department of origin, oxygen delivery method, NYHA cardiac function classification, and major comorbidities. Vital signs during the ultrasound examination were recorded, comprising respiratory rate, heart rate, systolic blood pressure, diastolic blood pressure, fraction of inspired oxygen, and oxygen saturation.

### 2.5 Statistical analysis

Statistical analysis were performed using SPSS, version 21.0 software (IBM Corp., Armonk, NJ, USA). The normality of continuous variables was assessed using the Kolmogorov–Smirnov test. Normally distributed continuous variables were expressed as mean ± standard deviation (SD), with intergroup comparisons conducted using independent samples *t*-test for two groups and one-way analysis of variance (ANOVA) followed by LSD-t test for multiple comparisons among three groups. Non-normally distributed continuous variables were presented as median [interquartile range (IQR)], analyzed by Mann–Whitney U test for two-group comparisons and Kruskal–Wallis test for three-group comparisons. Categorical variables were expressed as [*n* (%)], with *χ*^2^ test employed for intergroup comparisons. Binary logistic regression analysis was performed with variables from the univariable analysis (*P* < 0.1) to identify independent influencing factors. The predictive value was evaluated using receiver operating characteristic (ROC) curve analysis. Correlation analysis was conducted using Spearman's method, with the absolute value of coefficient r interpreted as follows: very strong (0.8–1.0), strong (0.6–0.8), moderate (0.4–0.6), weak (0.2–0.4), and very weak or no correlation (0–0.2). A two-tailed *P*-value < 0.05 was considered to indicate statistical significance. Intraclass Correlation Coefficient (ICC) were calculated and classified as poor (ICC < 0.40), weak (ICC = 0.40–0.59), good (ICC = 0.60–0.74), and excellent (ICC = 0.75–1.00).

## 3 Results

### 3.1 Baseline characteristics of the study population

The study initially included 751 patients presenting with acute dyspnea who underwent both echocardiography and lung ultrasound examinations, among whom 196 met the diagnostic criteria for CHF and inclusion criteria; after excluding 63 cases (including 4 with CT-confirmed pulmonary tumors, 3 newly diagnosed acute myocardial infarction, 3 congenital structural heart disease, 3 coma with absent spontaneous respiration, 3 pulmonary interstitial fibrosis, 2 acute exacerbation of chronic obstructive pulmonary disease (COPD), 2 prior heart valve replacement, 1 sepsis-induced cardiac dysfunction, 1 dilated cardiomyopathy, 1 thoracic surgery history, 8 inadequate ultrasound images or incomplete records, 26 non CPE or pneumonia pulmonary diagnoses, 4 undetermined final diagnoses, and 2 duplicate enrollments) **(**Figure 1**)**, the final analysis included 133 participants (77 males and 56 females), with an additional 30 subjects having CHF history without dyspnea selected from contemporaneous physical examinations or outpatient follow-ups were included in the CHF-control group (Table 1).

**Table 1:** Basic clinical characteristics of research subjects.

Parameter	CHF-CPE group (*n* = 58)	CHF-pneumonia group (*n* = 75)	CHF-control group (*n* = 30)	*P*-value
Age, years	73.00 ((61.75, 77.50)	72.00 (60.00, 81.00)	68.00 (61.75, 75.50)	0.291
Male, *n*(%)	29 (50.0%)	48 (64.0%)	17 (56.7%)	0.267
Department source, *n*(%)				<0.001
Emergency/Intensive Care Unit	8 (13.8%)	12 (16.0%)	0 (0)	
Cardiovascular medicine	25 (43.1%)	39 (52.0%)	18 (60.0%)	
Respiratory medicine	2 (3.4%)	9 (12.0%)	0 (0)	
Nephrology medicine	15 (25.9%)	13 (17.3%)	2 (6.7%)	
Other internal or surgical medicine	7 (12.1%)	2 (2.7%)	2 (6.7%)	
Other outpatient	1 (1.7%)	0 (0)	8 (26.7%)	
Respiratory Support, *n*(%)				0.682
Spontaneous breath	10 (17.2%)	9 (12.0%)	**–**	
Nasal cannula/mask oxygen inhalation	42 (72.4%)	57 (76.0%)	**–**	
High flow oxygen therapy/mechanical ventilation	6 (10.3%)	9 (12.0%)	**–**	
New York Heart Association				<0.001
Class II	5 (8.6%)	13 (17.3%)	11 (36.7%)	
Class III	28 (48.3%)	29 (38.7%)	19 (63.3%)	
Class IV	25 (43.1%)	33 (44.0%)	0 (0)	
Comorbidities, *n*(%)				
Coronary heart disease	43 (74.1%)	50 (66.7%)	21 (70.0%)	0.648
Hypertension	41 (70.7%)	47 (62.7%)	21 (70.0%)	0.573
Chronic kidney disease	21 (36.2%)	20 (26.7%)	5 (16.7%)	0.143
Cerebrovascular disease	2 (3.4%)	5 (6.7%)	1 (3.3%)	0.637
Diabetes	15 (25.9%)	15 (20.0%)	7 (23.3%)	0.723
Respiratory rate, times/min	22.50 (21.00, 25.25)	22.00 (21.00, 25.00)	19.00 (18.00, 19.25)^a^^,^^b^	<0.001
Heart rate, beats/min	85.00 (74.00, 98.00)	80.00 (72.00, 92.00)	76.00 (62.50, 86.00)^a^^,^^b^	0.011
Systolic blood pressure, mm Hg	134.43 ± 23.69	127.20 ± 20.09	130.27 ± 21.68	0.167
Diastolic blood pressure, mm Hg	76.64 ± 13.94	71.85 ± 12.33^a^	74.17 ± 10.96	0.011
Fraction of inspired oxygen	0.29 (0.29, 0.33)	0.29 (0.29, 0.33)	0.21 (0.21, 0.21)^a^^,^^b^	<0.001
Oxygen saturation, %	96.00 (91.99, 98.00)	96.34 (93.49, 98.00)	98.75 (96.58, 99.00)^a^^,^^b^	<0.001

a *P* < 0.05, compared with CHF-CPE group.

b *P* < 0.05, compared with CHF-pneumonia group.

### 3.2 Comparison of hemodynamic and echocardiographic parameters among the three groups

The CHF-CPE group demonstrated significantly higher values in IVCDmax, LAAmin, E velocity, E/A ratio, E/e′ ratio, TAPSE/MAPSE ratio, PASP, and ΔSV compared to both the CHF-pneumonia group and the CHF-control group (*P* < 0.05), while showing lower values in IVC-V, LAA-V, Septum e′, Lateral e′, and LVSV (*P* < 0.05). Additionally, the CHF-CPE group exhibited significantly lower MAPSE and LVOT-VTI values than the CHF-pneumonia group (*P* < 0.05). Compared to the CHF-control group, the CHF-CPE group had higher TRV but lower TAPSE/PASP ratio (*P* < 0.05). The CHF-pneumonia group showed significantly greater IVCDmax, TRV, and PASP values than the CHF-control group (*P* < 0.05). Furthermore, the CHF-CPE group had a significantly higher LUS score than the CHF-pneumonia group (*P* < 0.05). No statistically significant differences were observed in the remaining parameters among the groups (*P* > 0.05) (Table 2).

**Table 2:** Cardiopulmonary ultrasound indicators and cardiac coupled ultrasound indicators.

Parameter	CHF-CPE group (*n* = 58)	CHF-pneumonia group (*n* = 75)	CHF-control group (*n* = 30)	*P*-value
IVCDmax, mm	20.84 ± 3.94	17.41 ± 4.22^a^	15.38 ± 3.90^a^^,^^b^	<0.001
IVC-V, %	25.95 (19.97, 33.46)	43.88 (31.62, 53.25)^a^	44.17 (38.53, 56.81)^a^	<0.001
LAD, mm	44.00 (41.00, 47.00)	43.00 (40.00, 47.00)	45.50 (40.00, 48.00)	0.687
LAAmax, cm^2^	23.30 (20.30, 27.88)	22.10 (19.30, 24.60)	21.05 (19.28, 24.03)	0.054
LAAmin, cm^2^	18.60 (16.00,22.93)	15.90 (13.20, 18.70)^a^	14.20 (12.75, 17.63)^a^	<0.001
LAA-V, %	21.53 (15.73, 25.59)	28.75 (22.07, 33.47)^a^	30.14 (28.05, 33.59)^a^	<0.001
LVEDV, mL	132.50 (111.75, 161.75)	124 (97.00, 154.00)	124 (107.00, 154.00)	0.570
E, cm/s	113.00 (96.00, 128.25)	83.00 (69.00, 104.00)^a^	70.00 (63.75, 90.25)^a^	<0.001
A, cm/s	70.00 (42.00, 101.00)	67.00 (49.00, 93.00)	60.00 (40.00, 85.25)	0.453
Septal e’, cm/s	5.00 (3.98, 5.83)	5.20 (4.60, 6.30)^a^	5.95 (4.65, 7.00)^a^	0.002
Lateral e’, cm/s	7.05 (6.00, 8.00)	8.20 (7.00, 9.00)^a^	8.35 (7.18, 9.95)^a^	<0.001
E/A	1.61 (1.14, 2.55)	1.34 (0.78, 1.98)^a^	1.27 (0.64, 2.13)^a^	0.019
E/e’	19.36 (16.81, 22.32)	13.18 (10.80, 15.42) ^a^	10.16 (8.62, 12.94)^a^	<0.001
LVEF, %	46.00 (37.00, 59.25)	47.00 (39.00, 59.00)	48.00 (39.00, 59.00)	0.925
TAPSE, mm	19.10 (16.28, 21.53)	17.50 (15.50, 20.90)	18.35 (15.60, 20.08)	0.433
MAPSE, mm	9.40 (7.80,11.65)	11.90 (9.10, 14.30)^a^	10.45 (8.90, 13.80)	0.005
TAPSE/MAPSE	1.94 (1.62, 2.37)	1.58 (1.31, 1.86)^a^	1.81 (1.37, 1.90)^a^	<0.001
RV-s', cm/s	12.65 (9.95, 15.00)	11.30 (10.10, 14.10)	12.15 (9.83, 14.23)	0.712
TRV, m/s	3.00 (2.80, 3.30)	2.80 (2.40, 3.30)	2.45 (2.08, 2.80)^a^^,^^b^	0.001
PASP, mm Hg	48.64 (39.36, 57.39)	41.44 (31.04, 53.44)^a^	31.60 (24.12, 39.36)^a^^,^^b^	<0.001
LVOT-VTI, cm	13.45 (10.88, 17.38)	16.20 (13.40, 19.40)^a^	14.15 (12.30, 20.33)	0.004
RVOT-VTI, cm	14.25 (12.10, 19.10)	15.00 (12.50, 17.90)	13.85 (10.90, 18.98)	0.604
RVSV, mL	70.17 (61.03, 85.82)	69.18 (57.31, 82.75)	65.46 (49.12, 85.99)	0.529
LVSV, mL	53.07 (41.55, 63.78)	65.08 (53.69, 75.07)^a^	66.80 (52.07, 86.91)^a^	<0.001
ΔSV, mL	17.50 (10.29, 28.70)	5.59 (−4.20, 13.50) ^a^	−2.67 (−6.45, 5.15)^a^	<0.001
LUS score, score	14.00 (10.00, 18.00)	10.00 (7.00, 14.00)	**–**	<0.001

a *P* < 0.05, compared with CHF-CPE group.

b *P* < 0.05, compared with CHF-pneumonia group.

IVCD, inferior vena cava diameter; IVC-V, variation rate of inferior vena cava; LAD, left atrial diameter; LAA, left atrial area; LAA-V, variation of left atrial area; LVEDV, left ventricular end diastolic volume; E, early diastolic peak flow velocities of the mitral valve; A, late diastolic peak flow velocities of the mitral valve; e′, peak diastolic velocities of the mitral annulus; LVEF, left ventricular ejection fraction; TAPSE, tricuspid annular plane systolic excursion; MAPSE, mitral annular plane systolic excursion; RV-s’, right ventricular s’; TRV, tricuspid regurgitation velocity; PASP, pulmonary artery systolic pressure; LVOT-VTI, left ventricular outflow tract velocity time integral; RVOT-VTI, right ventricular outflow tract velocity time integral; RVSV, right ventricular stroke volume; LVSV, left ventricular stroke volume; ΔSV, The difference value between right ventricular stroke volume and left ventricular stroke volume; LUS, lung ultrasound.

### 3.3 Area under the ROC curve

#### 3.3.1 Comparisons between the CHF-CPE and CHF-pneumonia groups

**Figure 3:**
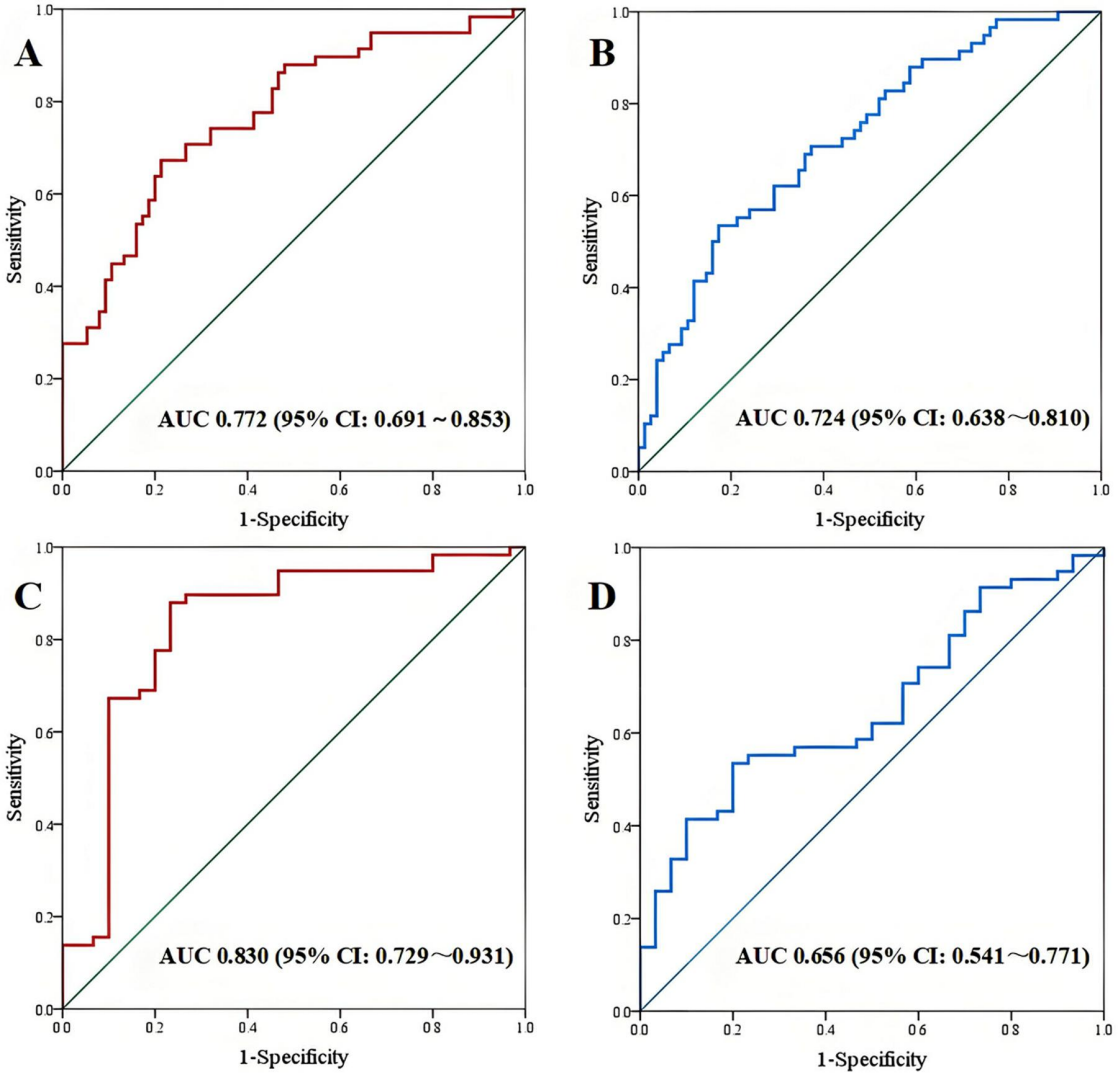
ROC curve. **(A,B)** AUC of ΔSV and TAPSE/MAPSE between CHF-CPE group and CHF-pneumonia group; **(C,D)** AUC of ΔSV and TAPSE/MAPSE between CHF-CPE group and CHF-control group.

In cardiac coupling ultrasound parameters, ΔSV demonstrated the highest diagnostic performance with an area under the ROC curve (AUC) of 0.772 (95% CI: 0.691–0.853), sensitivity of 67.24%, and specificity of 78.67%. TAPSE/MAPSE showed an AUC of 0.724 (95% CI: 0.638–0.810), with sensitivity of 53.45% and specificity of 82.67% (Figures 3A,B and Table 3). In other single parameters, E/e′ showed an AUC of 0.901 (95% CI: 0.850–0.952), sensitivity of 84.48%, and specificity of 84.00%. LAA-V demonstrated an AUC of 0.759 (95% CI: 0.680–0.839), sensitivity of 50.67%, and specificity of 91.38%.

**Table 3:** Diagnostic efficacy of ROC curve between CHF-CPE group and CHF-pneumonia group.

Parameter	Cut-off value	Sensitivity	Specificity	Yoden index	AUC	95% CI	*P*-value
ΔSV (mL)	14.129	67.24	78.67	0.459	0.772	0.691–0.853	<0.001
TAPSE/MAPSE	1.930	53.45	82.67	0.361	0.724	0.638–0.810	<0.001
E/e’	15.980	84.48	84.00	0.698	0.901	0.850–0.952	<0.001
LAA-V (%)	28.696	50.67	91.38	0.421	0.759	0.680–0.839	<0.001
LUS score (score)	13.500	62.07	74.67	0.368	0.734	0.650–0.818	<0.001

ΔSV, The difference value between right ventricular stroke volume and left ventricular stroke volume; TAPSE/MAPSE, the ratio of tricuspid annular plane systolic excursion to mitral annular plane systolic excursion; E/e’, the ratio of early diastolic peak flow velocities of the mitral valve to peak diastolic velocities of the mitral annulus at septal and lateral; LAA-V, variation of left atrial area; LUS, lung ultrasound.

#### 3.3.2 Comparisons between the CHF-CPE and CHF-control groups

In cardiac coupling parameters, ΔSV showed superior diagnostic performance with an AUC of 0.830 (95% CI: 0.729–0.931), sensitivity of 87.93%, and specificity of 76.67%. The TAPSE/MAPSE showed an AUC of 0.656 (95% CI: 0.541–0.771), sensitivity of 53.45%, and specificity of 80.00% (Figures 3C,D and Table 4). In other single parameters, E/e' showed an AUC of 0.918 (95% CI: 0.842–0.994), sensitivity of 96.55%, and specificity of 80.00%. LAA-V showed an AUC of 0.900 (95% CI: 0.835–0.964), sensitivity of 93.33%, and specificity of 72.41%.

**Table 4:** Diagnostic efficacy of ROC curve between CHF-CPE group and CHF-control group.

Parameter	Cut-off value	Sensitivity	Specificity	Yoden index	AUC	95% CI	*P*-value
ΔSV (mL)	5.039	87.93	76.67	0.646	0.830	0.729–0.931	<0.001
TAPSE/MAPSE	1.917	53.45	80.00	0.334	0.656	0.541–0.771	0.017
E/e’	13.043	96.55	80.00	0.766	0.918	0.842–0.994	<0.001
LAA-V (%)	24.565	93.33	72.41	0.657	0.900	0.835–0.964	<0.001

SV, The difference value between right ventricular stroke volume and left ventricular stroke volume; TAPSE/MAPSE, the ratio of tricuspid annular plane systolic excursion to mitral annular plane systolic excursion; E/e′, the ratio of early diastolic peak flow velocities of the mitral valve to peak diastolic velocities of the mitral annulus at septal and lateral; LAA-V, variation of left atrial area; LUS, lung ultrasound.

### 3.4 Univariate and multivariable logistic regression analysis

#### 3.4.1 Comparisons between the CHF-CPE and CHF-pneumonia groups

The multivariable logistic regression analysis revealed that ΔSV (OR: 1.076, 95% CI: 1.019–1.137, *P* = 0.008), E/e’ (OR: 1.663, 95% CI: 1.301–2.125, *P* < 0.001), LAA-V (OR: 0.837, 95% CI: 0.741–0.944, *P* = 0.004), and IVC-V (OR: 0.935, 95% CI: 0.888–0.985, *P* = 0.012) were independent risk factors for CPE in CHF patients (Table 5).

**Table 5:** Univariate and multivariable logistic regression analysis between CHF-CPE group and CHF-pneumonia group.

Factors	Univariate analysis			Multivariate analysis		
	Odds Ratio	95%CI	*P*-value	Odds Ratio	95%CI	*P*-value
IVCD	1.231	1.116–1.359	<0.001			
IVC-V	0.925	0.897–0.954	<0.001	0.935	0.888–0.985	0.012
LAAmin	1.101	1.023–1.185	0.011			
LAA-V	0.860	0.808–0.915	<0.001	0.837	0.741–0.944	0.004
E/A	1.809	1.198–2.733	0.005			
E/e’	1.667	1.400–1.984	<0.001	1.663	1.301–2.125	<0.001
TAPSE/MAPSE	6.114	2.589–14.435	<0.001			
LVSV	0.961	0.940–0.983	0.001			
ΔSV	1.081	1.046–1.117	<0.001	1.076	1.019–1.137	0.008
LUS score	1.218	1.116–1.330	<0.001			

IVCD, inferior vena cava diameter; IVC-V, variation rate of inferior vena cava; LAD, left atrial diameter; LAAmin, minimum left atrial area; LAA-V, variation of left atrial area; E/A, the ratio of early diastolic peak flow velocities of the mitral valve to late diastolic peak flow velocities of the mitral valve; E/e′, the ratio of early diastolic peak flow velocities of the mitral valve to peak diastolic velocities of the mitral annulus at septal and lateral; TAPSE/MAPSE, the ratio of tricuspid annular plane systolic excursion to mitral annular plane systolic excursion; LVSV, left ventricular stroke volume; ΔSV, The difference value between right ventricular stroke volume and left ventricular stroke volume; LUS, lung ultrasound.

#### 3.4.2 Comparisons between the CHF-CPE and CHF-control groups

The multivariable regression analysis demonstrated that ΔSV (OR: 1.066, 95% CI: 1.007–1.129, *P* = 0.027), E/e′ (OR: 1.475, 95% CI: 1.161–1.874, *P* = 0.001), and LAA-V (OR: 0.773, 95% CI: 0.635–0.941, *P* = 0.010) were independently associated with CPE in CHF patients (Table 6).

**Table 6:** Univariate and multivariable logistic regression analysis between CHF-CPE group and CHF-control group.

Factors	Univariate analysis			Multivariate analysis		
	Odds Ratio	95%CI	*P*-value	Odds Ratio	95%CI	*P*-value
IVCD	1.421	1.218–1.658	<0.001			
IVC-V	0.888	0.845–0.934	<0.001			
LAAmin	1.233	1.083–1.404	0.002			
LAA-V	0.710	0.608–0.829	<0.001	0.773	0.635–0.941	0.010
E/e’	1.659	1.353–2.035	<0.001	1.475	1.161–1.874	0.001
TAPSE/MAPSE	4.277	1.356–13.488	0.013			
LVSV	0.966	0.944–0.989	0.004			
ΔSV	1.085	1.043–1.129	<0.001	1.066	1.007–1.129	0.027
TAPSE/PASP	0.018	0.002–0.223	0.002			

IVCD, inferior vena cava diameter; IVC-V, variation rate of inferior vena cava; LAD, left atrial diameter; LAAmin, minimum left atrial area; LAA-V, variation of left atrial area; E/A, the ratio of early diastolic peak flow velocities of the mitral valve to late diastolic peak flow velocities of the mitral valve; E/e′, the ratio of early diastolic peak flow velocities of the mitral valve to peak diastolic velocities of the mitral annulus at septal and lateral; TAPSE/MAPSE, the ratio of tricuspid annular plane systolic excursion to mitral annular plane systolic excursion; LVSV, left ventricular stroke volume; ΔSV, The difference value between right ventricular stroke volume and left ventricular stroke volume; LUS, lung ultrasound.

### 3.5 Correlation analysis

**Figure 4:**
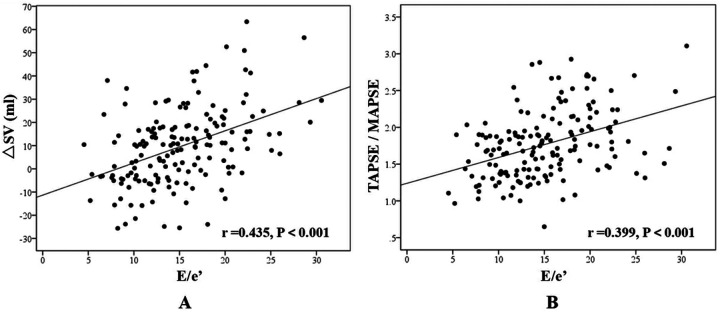
Correlation analysis. **(A)** The correlation between ΔSV and E/e′; **(B)** The correlation between TAPSE/MAPSE and E/e′.

Correlation analysis demonstrated a moderate positive correlation between △SV and E/e′ (*r* = 0.435, *P* < 0.001), while TAPSE/MAPSE showed a weak positive correlation with E/e′ (*r* = 0.399, *P* < 0.001) (Figure 4).

### 3.6 Repeatability test in intra- and inter-researcher

The study showed an excellent consistency of LVOT-VTI, RVOT-VTI, LVSV and RVSV in intra-researcher (ICC = 0.981, 0.929, 0.934 and 0.924, *P <* 0.001), and an excellent consistency of LVOT-VTI, RVOT-VTI, LVSV and RVSV in inter-researchers between the two ultrasound physician (ICC = 0.889, 0.860, 0.906 and 0.878, *P <* 0.001) (Table 7).

**Table 7:** Repeatability test in intra-researcher and inter-researchers (ICC).

Factors	LVOT-VTI	RVOT-VTI	LVSV	RVSV
intra-researcher *n* = 17	0.981 (0.949–0.993)	0.929 (0.818–0.973)	0.934 (0.831–0.975)	0.924 (0.808–0.972)
	*P* < 0.001	*P* < 0.001	*P*v0.001	*P* < 0.001
inter-researchers *n* = 17	0.889 (0.709–0.959)	0.860 (0.663–0.946)	0.906 (0.756–0.965)	0.878 (0.703–0.954)
	*P* < 0.001	*P* < 0.001	*P* < 0.001	*P* < 0.001

ICC, Intraclass Correlation Coefficient.

## 4 Discussion

CPE is the primary cause of acute dyspnea leading to hospitalization in CHF patients due to worsening cardiac function. However, long-term CHF patients may also develop pneumonia, which was not uncommon during the COVID-19 pandemic. CHF patients with pneumonia may exhibit an enlarged left heart and reduced LV function (including both systolic and diastolic dysfunction) on ultrasound, along with B-lines in unilateral or bilateral lung tissue on LUS. A history of CHF and typical ultrasound findings may mislead physicians to attribute B-lines to CPE, thereby prompting a decision to administer dehydration therapy. The essence of B-lines in LUS is pulmonary interstitial pathology, which can occur in pulmonary edema, pneumonia, alveolar hemorrhage, or pulmonary fibrosis. When using LUS to differentiate between CPE and pneumonia, the distribution pattern of B-lines is often key: CPE typically presents with diffuse B-lines in both lungs, whereas pneumonia manifests as irregular unilateral or bilateral focal B-line patterns. However, accurate differentiation becomes challenging when pneumonia diffusely involves the entire lung. In such cases, cardiac ultrasound plays a crucial diagnostic role.

Our study found that the CHF-CPE group exhibited significant differences in multiple cardiac ultrasound parameters compared to both the pneumonia and control groups, with the primary distinctions lying in left heart indicators. This observation aligns with previous studies (13, 14), which reported that patients with heart failure-induced pulmonary edema typically present with an enlarged left ventricle and impaired LV function. The contribution of E/e′ to extravascular lung water accumulation was greater than that of LVEF, a finding also supported by prior studies on mechanical ventilation weaning (4, 5). Elevated E/e′ levels are associated with higher LV filling pressures, reflecting reduced tolerance to fluid overload within the cardiopulmonary circulation.

Hemodynamically induced pulmonary edema is typically described as resulting from increasing LV filling pressures due to LV failure, leading to elevated pulmonary capillary hydrostatic pressure and subsequent fluid extravasation into the pulmonary interstitium. LV end-diastolic pressure (LVEDP) is generally considered the determinant of left atrial pressure and pulmonary venous pressure, which further governs pulmonary capillary pressure, ultimately contributing to acute pulmonary edema. However, isolated LV failure and elevated LV filling pressures alone do not entirely dictate the development of pulmonary edema. Clinically, we have observed that some patients with severe LV dysfunction exhibit no pulmonary fluid accumulation, whereas others with seemingly milder LV impairment (e.g., heart failure with preserved ejection fraction, HFpEF) present with significant pulmonary congestion. This phenomenon suggests that the development of pulmonary edema depends not only on LV pressure but also on sufficient SV from the RV, which serves as another critical factor (15). If the SV generated by RV contraction cannot be fully accommodated by the left atrium, excess fluid accumulates in the pulmonary circulation and interstitium (16). Conversely, impaired RV function is associated with worse clinical outcomes in CHF patients (17).

In the physiological state, the SV of RV and LV are maintained in balance through the Frank-Starling mechanism, ensuring pulmonary circulation homeostasis (6). For patients with pulmonary edema exhibiting an imbalance between RVSV and LVSV, hemodynamic monitoring is essential (18). Conventional imaging modalities for assessing RVSV and LVSV include computed tomography (CT) and magnetic resonance imaging (MRI) (19, 20). However, these techniques are costly and inconvenient, requiring patient transport to imaging departments—a particular challenge for CHF patients experiencing dyspnea. Given the safety and convenience of ultrasound technology, we should focus on the disparity between RV and LV parameters, which may better indicate the risk of pulmonary edema in CHF patients presenting with respiratory distress.

In this study, the CHF-CPE group demonstrated significantly higher E/e' ratios and lower LVOT-VTI and LVSV values (*P* < 0.05), while no statistically significant differences in LVEF were observed among the three groups (*P* > 0.05). These findings suggest that LV function, particularly impaired diastolic function and reduced SV, plays a crucial role in the development of pulmonary edema. In contrast, LVEF, which reflects the percentage of LV contraction and myocardial systolic performance, showed no significant correlation with pulmonary edema formation. Regarding RV parameters, echocardiographic indices including TAPSE, RV-s', RVOT-VTI, and RVSV showed no statistically significant differences among the groups (*P* > 0.05). This indicates that CHF patients with concurrent CPE maintain relatively preserved RV function, which can result in a mismatch between the right and left heart when LV function is reduced. Furthermore, elevated E/e′ ratios, typically indicative of increased LV filling pressure, may depend on adequate SV provision by the RV (21).

In this study, we employed echocardiography to quantify the SV difference between the RV and LV. The VTI and outflow tract cross-sectional area were measured at both the RVOT and LVOT to calculate respective SV. The ROC analysis revealed that the ΔSV difference demonstrated discriminative value between CHF-CPE and CHF-pneumonia groups, with an AUC of 0.772. Multivariate analysis identified this parameter as an independent predictor for CPE development in CHF patients (OR = 1.076, *P* = 0.008). Similarly, the ΔSV difference showed stronger discriminatory power between CHF-CPE and CHF-control groups (AUC = 0.830). This measure remained an independent risk factor for CPE (OR = 1.066, *P* = 0.027). These findings align with established hemodynamic theories regarding acute pulmonary edema development in heart failure patients (6, 15).

We introduced the TAPSE/MAPSE ratio as a novel indicator to assess the differential contractile function between the right and left ventricles. TAPSE and MAPSE represent the longitudinal displacement of the RV and LV free walls, respectively. In our cohort, the TAPSE/MAPSE demonstrated discriminative capacity between CHF-CPE and CHF-pneumonia groups, yielding an AUC of 0.724. When comparing CHF-CPE with CHF-control groups, the ratio showed an AUC of 0.656. In clinical practice, we often employ qualitative “eye-balling” assessments during echocardiography. Notably, we observed that some pulmonary edema patients exhibited more pronounced longitudinal motion in the RV free walls compared to the LV free walls, particularly in the apical four-chamber view. The TAPSE/MAPSE ratio provides quantitative validation of these visual observations. These findings align with the results reported by Zhang et al. (12), who demonstrated that TAPSE/MAPSE achieved an AUC of 0.761 (sensitivity 62.8%, specificity 77.9%) for predicting CPE in ICU patients. The key distinction of our study lies in its exclusive focus on CHF patients, whereas Zhang's cohort included ICU admissions that potentially comprised cases without underlying cardiac pathology or chronic ventricular dysfunction.

Correlation analysis revealed significant positive associations between both ΔSV and TAPSE/MAPSE ratio with E/e′ across all CHF cases (*r* = 0.435 and 0.399 respectively, *P* < 0.001). E/e′, as a surrogate marker of LV filling pressure, demonstrated strong correlation with pulmonary edema development. Additionally, we introduced a novel echocardiographic parameter, LAA-V (left atrial area variation), which was significantly reduced in the CHF-CPE group. Measured in the apical four-chamber view, LAA-V quantifies the percentage change in left atrial area from maximal to minimal dimensions during the cardiac cycle, reflecting the efficiency of atrial emptying into the LV. Lower LAA-V values correlated with greater pulmonary venous congestion. While left atrial volume variation might provide more comprehensive assessment, our study faced technical challenges in obtaining optimal image quality, particularly in the apical two-chamber view, due to: (1) suboptimal echocardiographic windows in acutely dyspneic CHF patients, and (2) Time constraints imposed by urgent clinical management. We recommend future investigations to evaluate left atrial volume variation parameters for more precise hemodynamic assessment.

LUS demonstrates significant diagnostic value in differentiating CPE from pneumonia (22, 23). Characteristic sonographic findings reveal distinct patterns: CPE typically presents with bilateral, symmetrical B-lines (24), whereas pneumonia manifests as focal/multifocal B-lines with concomitant consolidation (25). When evaluating acute dyspnea, echocardiography provides crucial complementary information—the detection of LV dysfunction strongly suggests CPE (26–28). However, diagnostic challenges arise in CHF patients due to pre-existing cardiac remodeling (including ventricular dilation and systolic dysfunction). While LUS score effectively quantifies pulmonary deaeration, it shows limited specificity for distinguishing CPE from pneumonia. In our study, although the CPE group exhibited higher LUS scores than the pneumonia group, this parameter failed to emerge as an independent predictor. This likely reflects distinct distribution patterns: (1) CPE patients predominantly showed diffuse 1–2 point scores across all 12 lung areas. (2) Pneumonia patients typically presented with minimal B-lines or normal A-lines (0 points) in anterior areas of chest, and consolidation and/or pleural effusion (3 points) in gravity-dependent regions. Notably, pneumonia patients demonstrated considerable interindividual variability in scoring. Our findings underscore that integrated cardio-pulmonary ultrasound (combining echocardiography with LUS) provides superior diagnostic performance for respiratory differentiation in this population.

## 5 Limitations

First, this is a single-centre, retrospective, observational study, and therefore the results cannot be generalised beyond the background of the research. It is important to acknowledge that the findings may be influenced by retrospective bias, an inherent limitation of the retrospective design. Furthermore, The study cohort was recruited from diverse clinical departments, including emergency medicine, intensive care, as well as general wards (e.g., cardiology and respiratory departments) managing patients with acute-onset dyspnea. Although all diagnoses were confirmed by board-certified intensivists and cardiologists, potential interdepartmental variations in disease severity and management should not be overlooked. Third, a subset of patients had already received mechanical ventilation upon emergency department admission. Although ventilator settings were maintained at minimal supportive parameters during ultrasound examinations, the interaction between mechanical ventilation and strong spontaneous respiratory efforts may have influenced cardiopulmonary ultrasound measurements. Finally, the sample size in our study was limited, particularly in the control group. This limitation underscores the necessity for future large-scale prospective research to validate the efficacy of this technology.

## 6 Conclusion

In patients with CHF, echocardiography may aid in differentiating between CPE and pneumonia. Our retrospective study suggests that the difference between RVSV and LVSV provides modest but significant discriminative capacity. However, these findings, potentially influenced by the sample size, require prospective multicenter validation before this parameter can be considered for guiding clinical decision-making to mitigate the risks of misdiagnosis.

## Data Availability

The original contributions presented in the study are included in the article/Supplementary Material, further inquiries can be directed to the corresponding author.
