# Supplementary figures and images for: Comparison of the right–left ventricular stroke volume difference evaluated by echocardiography in patients with chronic heart failure complicated with cardiogenic pulmonary edema and pneumonia
Source: Front Cardiovasc Med. 2025 Dec 5;12:1693941. doi: 10.3389/fcvm.2025.1693941 (PMC12714921; doi:10.3389/fcvm.2025.1693941)

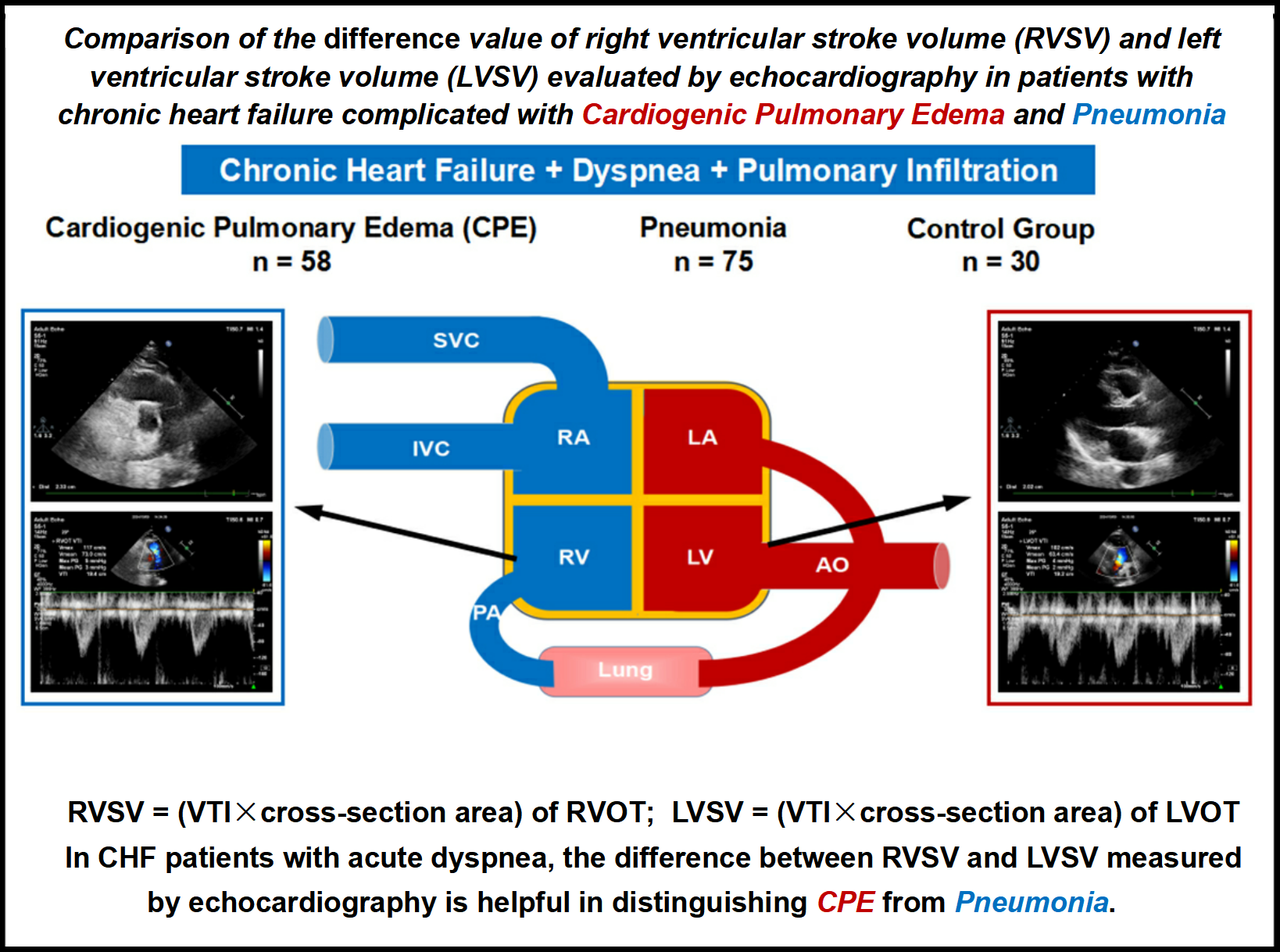

Supplement: Supplementary file 1 [file Image1.tif]
